# The interdisciplinary fracture liaison service improves health-related outcomes and survival of older adults after hip fracture surgical repair

**DOI:** 10.1007/s11657-022-01171-0

**Published:** 2022-10-17

**Authors:** Carmelinda Ruggiero, Marta Baroni, Giuseppe Rocco Talesa, Alessandro Cirimbilli, Valentina Prenni, Valentina Bubba, Luca Parretti, Riccardo Bogini, Giuliana Duranti, Auro Caraffa, Virginia Boccardi, Patrizia Mecocci, Giuseppe Rinonapoli

**Affiliations:** 1 Section of Gerontology and Geriatric, Department of Medicine and Surgery, S.M. Misericordia Hospital of Perugia, University of Perugia, S. Andrea Delle Fratte, Perugia, Italy; 2 Section of Orthopedics and Traumatology, Department of Medicine and Surgery, S.M. Misericordia Hospital, University of Perugia, Sant’Andrea Delle Fratte, Perugia, Italy; 3 Primary Care Physicians - SIMG, ASL 201, Perugia, Italy

**Keywords:** Fragility fractures, Fracture liaison service, Hip fracture, Osteoporosis, Anti-osteoporosis treatments

## Abstract

***Summary*:**

Due to the high burden of fragility fractures, we developed an interdisciplinary FLS care pathway for early management and monitoring of older adults discharged from a high-volume trauma center after hip fracture repair. Interdisciplinary FLS effectively improves up to 1-year adherence to treatments for secondary prevention of fragility fractures, reduces health facility admission, and improves long-term survival.

**Purpose:**

To compare adherence to secondary fragility fracture prevention, falls, healthcare facility admissions, and mortality between hip fracture older adults who entered the fracture liaison services pathway of care (FLS-CP) and those managed according to the usual traumatologist model of care (U-CP).

**Methods:**

Prospective observational study enrolling subjects aged ≥ 65 years discharged by high-volume trauma center after hip fracture repair from February 2016 to February 2017, who consecutively entered FLS-CP or U-CP according to their preference and goals.

**Results:**

Compared to U-CP, those in FLS-CP had higher initiation rate and up to 1-year adherence to secondary prevention of fragility fracture, including vitamin D and calcium (87.7% vs 36.9%; *p* < 0.0001), specific anti-osteoporosis drugs (75.1% vs 8.0%; *p* < 0.0001), and complete anti-fracture therapy (72.3% vs 5.7%; *p* < 0.0001). Older adults belonging to FLS-CP showed a lower likelihood of healthcare facility admission (RR 0.597; 95% CI 0.398–0.895; *p* = 0.0125), with a longer re-hospitalization-free survival (176.4 vs 88.7 days; *p* = 0.0152) than those in U-CP. One-year incidence of falls and fractures was similar between groups, with a lower tendency of the subjects in the FLS-CP to be multiple fallers (19% vs 34.8%; OR 0.057; 95% CI 0.004–0.876; *p* = 0.0690). The FLS-CP group experienced a lower 1-year (87.2% vs 74.3%; *p* = 0.001) and 3-year mortality (67.9% vs 55.6%; *p* = 0.0245) and a lower adjusted 5-year mortality hazard ratio (50.2% vs 58%; HR = 0.76; 95% CI 0.60; 0.96).

**Conclusion:**

The FLS-CP may improve initiation and adherence to secondary prevention of fragility fractures, reduces healthcare facility admission, and improves long-term survival.

**Supplementary Information:**

The online version contains supplementary material available at 10.1007/s11657-022-01171-0.

## Introduction

Falls and fragility fractures may hurt older adults’ quality of life and their families due to catastrophic loss of independence, morbidity, permanent disability, and death [1, 2]. Hip fractures are a priority among fragility fractures causing the most significant cost burden on healthcare services in Italy and worldwide [3].

In 2000, the incidence of hip fractures was about 1.6 million worldwide. Accounting for the aging population, authors estimate that hip fractures will reach 6.3 million in 2050 [4]. In Italy, hospitalizations due to hip fractures reached 94,525 in 2014, mainly affecting the oldest old subgroup, representing 2.5% of the population [5]. In the Umbria region, hospitalizations due to hip fractures were 1825 in 2014, with a peak of 1885 in 2012. The direct cost estimations reached € 18,566,633 in 2014 and peaked at € 19,177,043 in 2012 [5].

Several institutions strongly recommend interventions to prevent fragility fractures. Balancing vitamin D and calcium deficiency, encouraging physical activity, and a healthy lifestyle are priorities in the general population. Promoting initiation and adherence to appropriate anti-osteoporosis medications over the top of previous interventions is mandatory among high-risk subjects [6–8]. The cost-effective ratio of anti-osteoporosis treatments has been proved among high-risk patients, especially those with previous hip fractures [9, 10]. However, appropriately managing subjects at high risk of fragility fractures remains an unsolved issue with many barriers identified: uncertainties about who is responsible for osteoporosis care once a fracture has occurred and the lack of coordinated services with the capability to receive, assess, and monitor treatments of an increasing number of high-risk subjects [11].

Fracture liaison services (FLSs) are interdisciplinary or integrated clinical pathways particularly welcomed for providing care to high-risk fracture subjects [12, 13]. The FLSs may grant a synergy between in-hospital and community care services and professionals, overcoming patients’ issues and obstacles to diagnostic and therapeutic initiation. FLS has been identified among cost-effective interventions [13–16], mainly if an interdisciplinary service provides care [17, 18]. Besides interventions dedicated to patients’ and caregivers’ education, falls and fracture prevention, FLS may support a gaining in QALYs and optimize the country-specific cost per QALY [10]. However, high heterogeneity characterizes available FLSs, which are usually unique to the providers, needs, healthcare systems, and patients they serve. Direct comparisons are not feasible among FLS models. Outcomes mainly depend on the methodology adopted to implement the services, including the availability of dedicated resources, the adoption of performance indicators, and a quality improvement approach [13, 16–19].

FLSs are not an established pathway of care at the regional and national levels in our country, and further evidence may help make them considered a standard of care. We showed that implementing a FLS improved identification, clinical management, and adherence to anti-osteoporosis treatments in a high-volume trauma center [20]. We implemented the outpatient service to provide an interdisciplinary assessment at 30-day from the patient’s hospital discharge after hip fracture repair. The main novelty of the service is the contextual assessment by the orthopedic surgeon and geriatrician, providing patients with tailored interventions to optimize pharmacological treatments, functional recovery, and appropriate monitoring. We hypothesize that such a service could improve clinical outcomes of older adults who underwent hip repair and positively impact healthcare-related burden.

The primary aim of this study is to confirm the impact of an innovative interdisciplinary FLS care pathway (i.e., FLS-CP) as compared to a usual care pathway (i.e., U-CP) on patients’ adherence to timely prescribed anti-osteoporosis treatments and then to explore the service’s effect on healthcare service utilization, fall and fracture incidence, functional recovery, and mortality as secondary outcomes.

## Methods

### Study design and sample

A prospective observational study was conducted among subjects aged > 65 years, consecutively discharged from the academic hospital in Perugia, Italy, after surgical repair of hip fracture from February 2016 to February 2017. The hospital meets the acute care needs of almost 900,000 inhabitants (Umbria, Italy), of which 24% are over 65 years. Based on previous experience [20], the trauma and orthopedic unit and the fracture prevention service established an outpatient interdisciplinary service to provide a 30-day post-surgical visit to patients who underwent hip repair. Patients hospitalized due to hip fragility fractures received usual orthopedic care management mainly focused on surgical management of hip fracture and relative outcomes. Patients were scheduled to outpatient visit if their hip fracture was adjudicated a fragility fracture by the traumatologist. At the time of discharge, they received recommendations by the orthopedic surgeon to have a check of the surgical outcomes at 30 days from discharge. A dedicated administrative assistant handled lab and X-ray exams and scheduled the 30-day post-surgical visits by accessing the centralized hospital server. All patients received exam prescriptions and calendar appointments written in the discharge summary sent to patients’ general physicians (GPs).

During the 30-day visit, the traumatologist invited patients to enter the multidisciplinary service (FLS-CP). According to their preferences and goals, some patients opted to join the FLS-CP, receiving a traumatologist’s and geriatrician’s evaluation based on CGA, while others were satisfied with U-CP led by the traumatologist. Both groups received indications about the healing process, weight-bearing, thromboprophylaxis, exercise, and rehab program. Participants who entered the FLS-CP received in addition drug revision, diet advice, and fall and fracture prevention interventions. All patients returned the summary notes to their GPs who were involved in the process of care.

From June 2016 to April 2017, all participants received telephone follow-up calls at 3, 6, and 12 months from their individual baseline visit. A trained geriatrician conducted patient and caregiver interviews using standard approaches and scales. Patients who did not answer the first call received at least three more telephone calls. The regional administrative registry provided information about the vital status. The study was consistent with the Helsinki declaration’s ethical standards. The ethics committee of the regional healthcare system approved the study with registration number 2257/14.

### Data collection

Baseline participants’ data were gathered from clinical records, including demographics, type of fracture, time to surgery, type of surgery, weight-bearing, and pre-fracture functional level. Classification of hip fractures was consistent with the Orthopaedic Trauma Association [21]. Decisions about the type of surgical repair were made based on a consensus by two surgeons, with prosthetic replacement as the primary surgical indication for a medial fracture and osteosynthesis for a lateral femur fracture. Pre-fracture functional abilities were classified by using the basic Activity of Daily Living (BADL) score, ranging from 0 to 6 [22], and the Instrumental Activity of Daily Living (IADL) score, ranging from 0 to 8 [23]. Patients were classified as independent in BADL if they were able to perform at least five tasks. The IADL independence was defined according to gender-specific thresholds as women able to perform at least 6 of 8 and men able to perform at least 3 of 4 tasks [23]. Participants who entered FLS-CP received CGA [24], with additional information about anthropometric parameters (weight, height, and body mass index (BMI)); cognition, mood, and behavioral symptoms (Mini-Mental State Examination (MMSE) test, 5-item Geriatric Depression Scale (GDS5) test, Informant Questionnaire on Cognitive Decline in the Elderly (IQCODE) test, Clinical Dementia Rating (CDR) test, Mini Nutritional Assessment (MNA) test); comorbidities; polypharmacy; social-environmental aspects; and other risk factors, previous fractures, and bone fragility diagnostics and treatments.

Over the follow-ups, information was collected about ongoing treatments, comorbidity, functional recovery, performances, falls, fractures, and adverse events causing healthcare facility admission. Participants and their caregivers were asked about hospitalization, emergency room (ER) admissions, falls and fractures, and long-term care facility (LTC) admission. Participants receive specific questionnaires about adherence to vitamin D and calcium intake or supplements, bisphosphonates (BPs), antibodies against rank-ligand (denosumab), and parathyroid hormone (PTH) analogs. Data about the participants’ vital status come from the regional administrative issue released in March 2022.

### Primary and secondary outcomes

The primary endpoint was the patients’ adherence to anti-osteoporosis treatments initiated within 30 days from surgery. The secondary endpoints were re-hospitalization rate, days of hospitalization-free survival (HFS), ER admissions, rate of falls and fractures, LTC admission, and mortality. The healthcare burden was defined as a composite outcome taking into account the total rate of hospitalizations, ER, and LTC admissions.

### Statistical analyses

Categorical variables were reported as absolute and relative frequencies and continuous outcomes as mean ± standard deviation. The differences in baseline characteristics were tested using Wilcoxon test for continuous variables and Fisher exact test for categorical variables. Participants’ characteristics were summarized by the time they contributed to the follow-up: T1 includes data from participants who contributed up to 90 days; T2 and T3, those participants who contributed 91–210 days and 211–413 days, respectively, from the outpatient visit (T0), performed 30 days from index surgery. Besides, the differences between the groups were tested using generalized linear models (gamma regression for continuous variables, Poisson regression for recurrent events, and logistic regression for proportions), adjusting for age, sex, BADL, and IADL independence and duration of follow-up. The mean effect was expressed as mean absolute differences (Δ), odds ratio (OR), and rate ratio (RR) for continuous variables, dichotomous variables, and events, respectively. For the analyses, the 95% confidence interval (95% CI) was reported. Survival analysis was explored as a secondary outcome by using Kaplan–Meier curves. Hazard ratio (HR) was calculated using Cox regression analysis adjusting by age and sex. For all tests, α was set at 0.05; all *p*-values were two-sided. All the statistical analyses were performed using R software, 3.1.2 version.

## Results

### Baseline participants’ characteristics

**Fig. 1:**
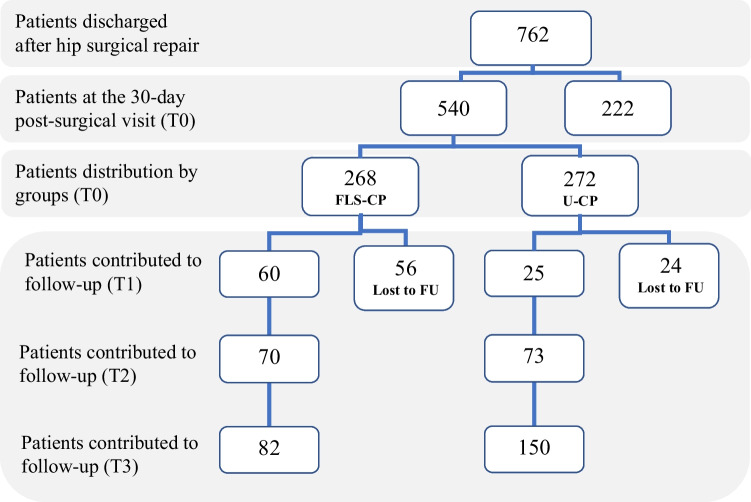
Description of the cohort at baseline and over 1-year follow-up

Figure 1 shows the distribution of the participants over the study period. Over 1 year, 762 patients were discharged after hip fracture repair and scheduled for a 30-day post-surgical outpatient visit: 75.5% were women, and mean age was 83.5 years, affected by medial hip fracture (43.7%) and lateral hip fracture (54.1%) (data not shown). Of them, 540 (71.0%) attended the visit (T0), with 272 (50.3%) entering the U-CP and 268 (49.6%) the FLS-CP, while 222 (21%) patients did not come back at the 30-day visit from discharge. Reasons for not attending the 30-day outpatient visit are missing: these patients did not answer the telephone calls over the entire follow-up period.

Participants who progressively entered the FLS-CP received follow-up calls up to 90 days (*n* = 106; 19.6%), from 91 to 210 days (*n* = 177; 32.8%), and 211 to 413 days (n = 257; 47.6%) from T0. Over time, 56 (20.8%) participants belonging to FLS-CP and 24 (8.8%) to U-CP did not answer the telephone calls and were treated as lost at the follow-up. Table 1 shows the main characteristics of participants by FLS-CP and U-CP groups. The majority of them were women (80.2% in FLS-CP versus 73.8% in U-CP), with a mean age of 83 years. More than half of the participants were independent in BADL before fracture (60.8% in FLS-CP versus 59.3% in U-CP; *p* = 0.7646), with 38.7% and 46.8% reported gender-specific IADL independence in the FLS-CP and U-CP group, respectively (*p* = 0.1121). Overall, pre-fracture IADL independence level was lower in the FLS-CP than U-CP group (*p* = 0.0042), especially among women, suggesting a higher burden of poor health in the former than the latter group.

**Table 1:** Participants’ characteristics at the 30-day post-surgical visit grouped by care pathway

	Total cohort		
	FLS-CP (*n* = 212)	U-CP (*n* = 248)	*p*-value
Women, *n* (%)	170 (80.2%)	183 (73.8%)	0.1213
Age, year (mean ± SD)	83.8 ± 7.4	83.7 ± 7.6	0.9492
Pre-fracture BADL, median score (IQR)	5 (3; 6)	5 (3; 6)	0.777
Pre-fracture BADL independence, *n* (%)	129 (60.8%)	121 (59.3%)	0.7646
Pre-fracture IADL, median score (IQR)			
- Overall	3 (0; 7)	4 (2; 7)	0.0042
- Women	3 (0; 7)	5 (2; 7)	0.1006
- Men	1.5 (0; 4)	4 (2; 8)	0.0037
Pre-fracture IADL independence, *n* (%)	82 (38.7%)	95 (46.8%)	0.1121
Hip fracture type, *n* (%)			0.3819
- Lateral	109 (56.8%)	125 (52.3%)	
- Medial	83 (43.2%)	114 (47.7%)	
Surgery, *n* (%)			0.5532
- Prosthesis	82 (42.7%)	93 (38.4%)	
- Osteosynthesis	110 (57.3%)	142 (58.7%)	
Time to surgery, day (mean ± SD)	4.1 ± 2.2	3.9 ± 1.9	0.4141
Surgery within 48 h, *n* (%)	44 (21.4%)	28 (12.0%)	0.0097
Weight-bearing, *n* (%)			< 0.001
- Early	176 (91.7%)	152 (76.8%)	
- Delayed	16 (8.3%)	46 (23.2%)	

*SD* standard deviation, *BADL* basic activities of daily living, *IADL* instrumental activities of daily living, *IQR* interquartile range

There was no difference in the type of fractures between groups (*p* = 0.3819). The trochanteric fractures (56.8% versus 52.3% in FLS-CP and U-CP groups, respectively) overcame femoral neck fractures (43.2% versus 47.7% in FLS-CP and U-CP groups, respectively). The less frequent AO categories were A3 among the trochanteric and B3 among the femoral neck fractures [21] (data not shown). Osteosynthesis was the most prevalent surgical treatment (57.3% in the FLS-CP and 58.7% in U-CP; *p* = 0.5532). The mean time to surgery was close to 4 days, similar between groups (*p* = 0.4141), with 21.4% and 12% of the participants belonging to the FLS-CP and U-CP groups receiving early surgery, respectively (*p* = 0.0097). The rate of early weight-bearing was higher in the FLS-CP compared to U-CP (91.7% versus 76.8%, respectively). Overall, one out three patients received delayed weight-bearing. Notably, the baseline characteristics of participants who contributed or lost during the follow-up in the FLS-CP group were similar. In contrast, participants who contributed to U-CP were younger (83.7 years) than those lost (87.6 years) over the follow-up (Online Resource 1). Then, we assumed that excluding older patients lost to follow-up in the U-CP group may attenuate the age effect on outcomes by comparing FLS-CP and U-CP groups.

Data gathered by geriatrician at the 30-day post-surgical visit (T0) by using CGA in patients entering the FLS-CP group showed median BMI 23 kg/m^2^ (interquartile range 21–26), with 60.2% at risk of malnutrition and 24.7% malnourished. CIRS comorbidity score was 7 (interquartile range 5–9), with high prevalence of low vision (41.5%) and poor hearing (34.9%), as well as hypertension (61.3%), arthrosis (34.9%), cardiac disease (26.4%), atrial fibrillation (17.5%), diabetes (15.1%), COPD (14.2%), depression (11.8%), cancer (10.4%), hypothyroidism (10.4%), and acute kidney injury (10.4%) (Online Resource 2). One out of three subjects (28.3%) reported one or more falls in the previous year. One out of four (24%) had moderate to severe cognitive impairment, and 9.4% had fragility fractures before the index event. Among fragility fractures, 11.4% were at the vertebral, 9.9% at the wrist, 10.3% at the rib, and 16.4% at other sites. Before index fracture, few patients were taking vitamin D (*n* = 31; 14.6%), calcium supplementation (*n* = 8; 3.8%), and BPs (*n* = 8; 3.7%) as specific anti-osteoporosis drug. At the time of the 30-day post-surgical visit, one out of three people (*n* = 60; 29%) had a DXA assessment, and 67.5% of them showed a T-score less than 2.5 SD at hip or vertebral site. Then, 97.6% (*n* = 207) initiated vitamin D and calcium supplementation if dietary intake was not sufficient, and 63.7% (*n* = 135) were prescribed complete anti-osteoporosis treatments. Main reasons for not initiating complete anti-osteoporosis treatment included vitamin D deficiency and incomplete blood test, especially those related to kidney function.

### Adherence to intervention for secondary prevention

**Fig. 2:**
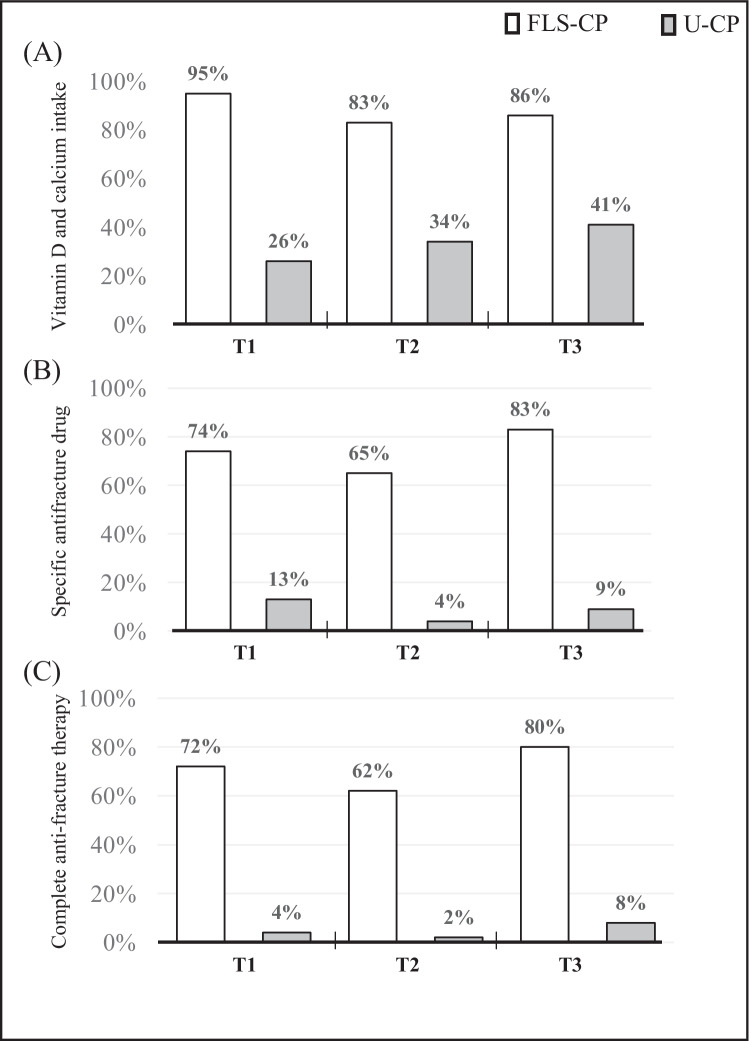
Participants’ adherence to treatments over the 1-year follow-up according to the care pathway. Note: The variable “Specific anti-osteoporosis drugs” includes bisphosphonates, teriparatide analog, and denosumab, and that “Complete anti-osteoporosis therapy” is defined as vitamin D plus adequate calcium intake and specific anti-osteoporosis drugs

Figure 2 shows patients’ medication adherence over follow-up times according to their contribution up to 90 days (T1), from 91 to 210 days (T2), and 211 to 413 days (T3) from baseline (T0). Compared to subjects in U-CP, those belonging to FLS-CP showed higher adherence to vitamin D supplementation plus calcium (Fig. 2A), to specific anti-osteoporosis drugs, i.e., BPs, denosumab, or PTH analogs (Fig. 2B), and complete anti-osteoporosis treatments (Fig. 2C) in all time points. Overtime, FLS-CP group had higher probability of being prescribed and taking vitamin D and calcium supplementation (87.7% vs 36.9%; OR = 13.6, 95% CI 7.7; 24.3), specific anti-osteoporosis drugs (75.1% vs 8.0%; OR = 56.0, 95% CI 25.5; 122.9), and complete anti-osteoporosis therapy (72.3% vs 5.7%; OR = 87.5, 95% CI 35.0; 218.6) than U-CP. The specific anti-osteoporosis drugs’ distribution was similar between groups: 47% were on BPs, 51.5% on denosumab, and 3–5% on PTH analogs (*p* > 0.9999). With regard to BPs treatments, the oral treatment overcomes the intravenous one (86% versus 14%, respectively), with patients’ reluctance to carry out infusion therapy (48%) on one side and lack of resources to assure timely BP infusion (38%) on the other side. Focusing on anti-osteoporosis treatments in the subgroup of patients receiving DXA at T0 (*n*: 60), we found that 88% were on complete treatment at T1, 65% at T2, and 91% at T3.

### Falls, fractures, and healthcare service utilization

Table 2 reports data about falls, fractures, and healthcare service utilization over the follow-up. Fall incidence rate was almost overlapping between the groups: 31.1/100 person-years in the FLS-CP and 24.4/100 person-years in the U-CP (RR = 1.021; 95% CI 0.607–1.717), as well as the fracture incidence 5.4/100 person-years in FLS-CP and 6.9/100 person-years in the U-CP (RR = 0.783; 95% CI 0.261–2.353). Compared to U-CP, there was a tendency to a lower prevalence of multiple fallers (19% versus 34.8%; OR 0.057; 95% CI 0.004–0.876; *p* = 0.0690) in the FLS-CP group, but the distribution of the absolute numbers of falls and fractures was similar between the groups (*p* = 0.8322). Overall, new fractures occurred at hip (28.6%), femur (26.8%), ankle (14.3%), wrist (14.3%), humerus (7.1%), and vertebra (7.1%).

**Table 2:** Adverse events and healthcare facility admission over 1-year follow-up

	FLS-CP	U-CP	FLS vs U-CP (95% CI)	*p*-value
Falls	31.1 (20.8; 44.6)	24.4 (16.7; 34.5)	*RR* = 1.021 (0.607; 1.717)	0.9366
- Multiple fallers (% patients)	19% (6.3; 42.6)	34.8% (17.2; 57.2)	*OR* = 0.057 (0.004; 0.876)	0.0399
Fractures	5.4 (1.7; 12.5)	6.9 (3.1; 13.0)	*RR* = 0.783 (0.261; 2.353)	0.6640
Health facility admissions*	40.7 (28.8; 55.9)	58.0 (45.7; 72.6)	*RR* = 0.597 (0.398; 0.895)	0.0125
- Hospital	18.2 (10.6; 29.1)	28.2 (19.9; 38.9)	*RR* = 0.606 (0.333; 1.102)	0.1005
- ER	16.1 (9; 26.5)	23.7 (16.1; 33.6)	*RR* = 0.574 (0.300; 1.096)	0.0925
- LTC facilities	6.4 (2.4; 14)	7.6 (3.7; 14.0)	*RR* = 0.686 (0.244; 1.930)	0.4754
HFS (days)	176.4 (89; 263)	88.7 (49.2; 128.2)	Δ = 85.8 (23.9; 147.7)	0.0152

*CI* confidence interval, *HFS* hospitalization free survival, *MF* multiple faller, *ER* emergency room, *LTC* long-term care, *RR* rate ratio, *Δ* absolute mean difference

Data are presented as events/100 person-years or otherwise specified in parenthesis

^*^Composite outcome taking into account the total rate of hospitalizations, ER admissions, and hospitalization in long-term care facilities

Health facility admissions were lower in the FLS-CP group (40.7/100 person-years) than in the U-CP group (58.0/100 person-years). Compared to the U-CP group, the participants belonging to the FLS-CP had a lower likelihood of healthcare facility admission (RR = 0.597; 95% CI 0.398–0.895; *p* = 0.0125). A similar trend occurred for the sub-components, more pronounced in the ER visits and less in the LTC facility admissions. The average time from the T0 visit to re-hospitalization was 176.4 (89–263) days in the FLS-CP group and 89 (49–128) days in the U-CP, with a difference of almost 3 months (Table 2). Overall, the leading causes of hospitalizations were pneumonia (22.2%), cerebrovascular events (16.7%), orthopedic revision and surgery (16.7%), anemia (9.3%), and cardiac events (9.3%), with similar distribution between the groups (*p* = 0.6587).

### Functional recovery and mortality

**Fig. 3:**
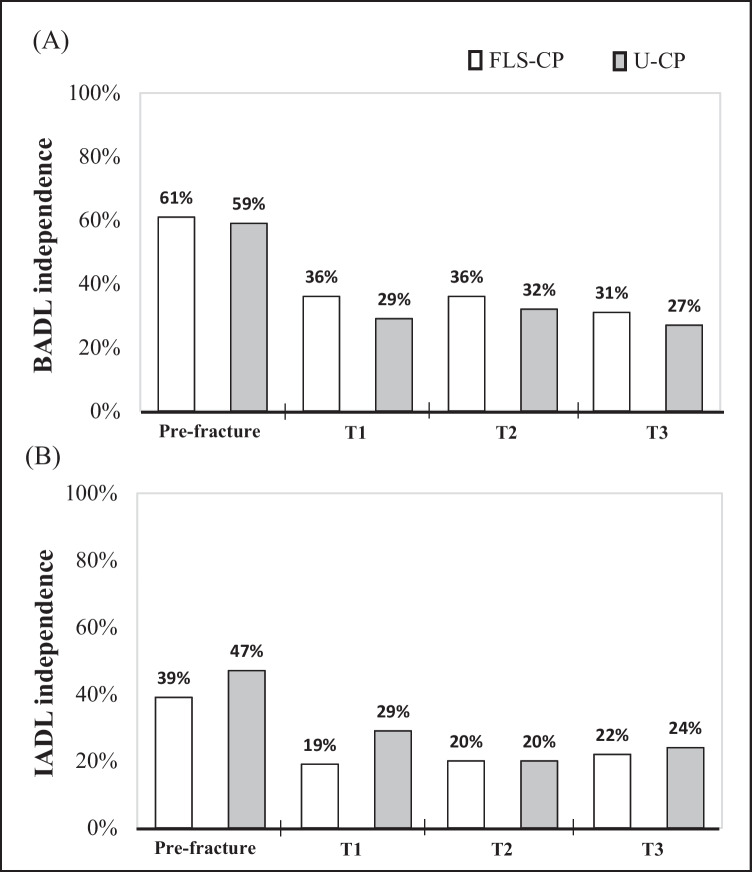
Participants’ functional status before and over 1-year after hip fracture surgical repair. BADL, basic activities of daily living; IADL, instrumental activities of daily living

Figure 3 presents the proportion of subjects with BADL and IADL independence over the follow-up time (Fig. 3). In both groups, most subjects were functionally independent before index fracture: 60% in BADL and 43% in IADL, but only 29% and 23% of them recovered independence after 1-year follow-up. Compared to U-CP, FLS-CP was associated with equivalent likelihood to recover BADL (OR = 1.66; 95% CI 0.87; 3.16) and IADL independence (OR = 1.00; 95% CI 0.50; 1.98).

**Fig. 4:**
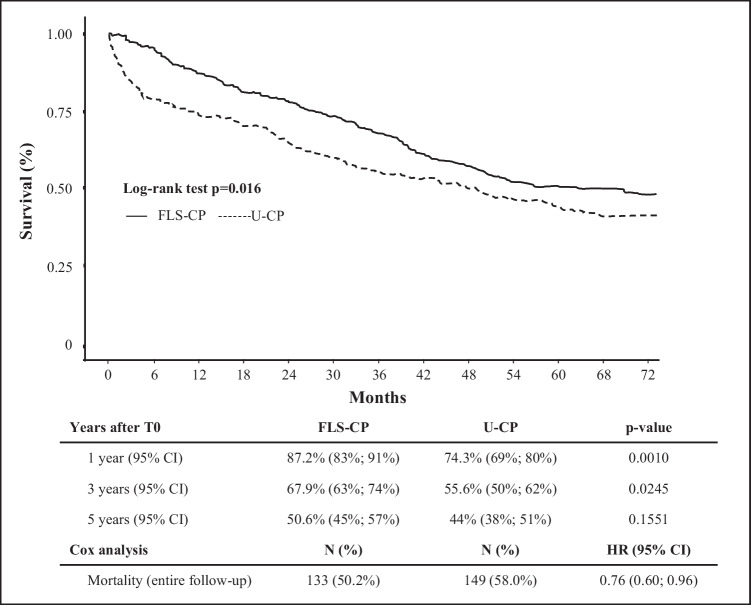
Kaplan–Meier curves for long-term survival after hip fracture repair according to care pathway. CI, confidence interval; HR, hazard ratio

Patients belonging to FLS-CP and the U-CP groups experienced 133 and 149 deaths over 5-year study period. Patients’ survival was 69 months in the FLS-CP and 49 months in the U-CP group (Fig. 4). According to Kaplan–Meier analysis, patients in the FLS-CP group showed higher survival than those in the U-CP group (*p* = 0.016), especially in the three years post-hip fracture repair (Fig. 4). Compared to the U-CP group, the survival advantage was higher in the FLS-CP group at 1 year (87.2% versus 74.3%; *p* = 0.0010) and at 3 years (67.9% versus 55.6%; *p* = 0.0245), with only a tendency (50.6% versus 44%; *p* = 0.1551) at 5-year from hip fracture surgery (Fig. 4). The Cox proportional hazard model showed a lower 5-year mortality in the FLS-CP group than in the U-CP independent of age and sex (HR 0.76, 95% CI 0.60; 0.96).

## Discussion

This study confirmed a higher adherence to secondary prevention fragility fractures of older adults with previous hip fractures who entered the FLS-CP than those who followed the U-PC. Although the absolute numbers of people entering and following the innovative pathway of care were lower than that observed in the usual care pathway, we found that about three out of four subjects belonging to the FLS-CP initiated appropriate treatments within 30 days from hip fracture surgery and remained on treatments for up to 1 year. Older adults in the FLS-CP group experienced a lower probability of being multiple fallers, without difference in fracture incidence over 1-year follow-up. However, subjects in the FLS-CP experienced lower health facility admissions and a higher hospital-free survival than those in the U-CP. Furthermore, older adults belonging to the FLS-CP experienced lower 5-year mortality than those in the U-CP group, with evidence for an early survival advantage in the FLS-CP than in the U-CP group.

Identifying interventions driven by the CGA is an essential pillar of the FLS-CP. Our service is a physician-led FLS offering patients’ evaluation by orthopedic surgeons and geriatricians and providing integrated notes to manage bone fragility and frailty of older adults with hip fracture repair [25, 26]. The integrated multidisciplinary approach delivers tailored interventions for fractures and fall prevention, taking into account optimization of comorbidity and polypharmacy, frailty, functional and social-behavioral aspects, patients’ preferences, and care goals [24–26]. The interdisciplinary approach may strengthen adherence to treatments, improve the awareness of the causal relationship between osteoporosis and fragility fractures by patients and caregivers, and even support the GPs in fostering persistence in the secondary prevention of fragility fractures [27–29]. However, there is a need for systematic and standardized implementation of such services especially among high-risk patients [13, 16]. We acknowledge the issues associated with entering the service based on patients’ care goals and preferences, perhaps under-powered the possible impact of the service on maximizing adherence to treatments among patients highly deserving them.

Similar to previous study, our FLS care pathway profits from a local network of available services, including an administrative assistant in charge of scheduling patients’ exams and visits [20, 30]. The administrative assistant resulted particularly helpful to overcome patients’ non-clinical, educational, and financial barriers, improving patients’ timing for entering the care pathway and reducing dispersion through the follow-up. Building on the local network of services, high-risk patients received lab exams and X-ray evaluation of repaired hips. One-third of FLS-CP recipients had BMD testing within 30 days from surgery, improving previous timing and performance [20]. However, DXA scans cannot be considered a marker of how often patients were worked-up for fracture risk. In most subjects, the secondary prevention of fragility fractures started independently of a T-score threshold [31].

A unique feature of our interdisciplinary FLS-CP is the capability to capture more than a half of patients deserving secondary prevention of fragility fractures in a high-volume trauma center. Although our service is still evolving to fulfill the leading key performance indicators [28], our performance is close to that of a French FLS service [32] and higher than those from previous other experiences [20, 30–36]. Both structural and process features might ensure the FLS satisfies the healthcare demands of high-risk patients [16, 17, 36] and contribute to the effectiveness of increasing patients’ adherence to timely initiated treatments. Over 70% of patients in the FLS-CP were on complete anti-fracture therapy 3 months later the post-surgical visit, and 80% persisted on treatments over the follow-up up to 1 year. In this context, it is conceivable that a higher rate of drug prescription or initiation of treatments close to the index event may increase medication adherence and persistence [33, 35]. We argue that interdisciplinary management might have been crucial to overcoming previous results showing that almost half of hip-fracture patients receive anti-osteoporosis drug prescriptions within 3 months from hip fracture repair, and 39% assured 1-year drug adherence [20].

Contrary to previous studies, we failed to demonstrate that FLS-CP reduces the rate of new fractures and falls within 1 year [37–39]. Gomez et al. showed that an integrated multidisciplinary care model reduces falls and fractures over 6 months [37]. Inderjeeth et al. proved that FLS minimizes the rate of new fractures [38]. González-Quevedo et al. explained the apparent failure of FLS in reducing the secondary fractures over 1 year since hip fracture based on the underdiagnosis of vertebral fractures, which are the most common in this subgroup [39]. Findings from our study support that vertebral fractures are frequently underdiagnosed due to patients’ lack or atypical symptoms [39, 40].

Falls are strongly associated with hip fractures. One out of two patients develops them within 1 year, and one in ten patients experiences further fractures [41]. Growing evidence supports bone-muscle interaction leading to sarcopenia and osteoporosis complex, high risk of falls, mobility disability, and fractures [42]. According to previous studies, a high vitamin D initiation and adherence rate may significantly prevent falls [43]. Combining vitamin D, tailored exercise, diet, and pharmacological advice might have impacted risk factors for falls, possibly reducing the proportion of multiple fallers, but there was no effect on falls in our study.

We add to the literature by showing that FLS reduces the rate of health facility admissions, including ER and re-hospitalization due to adverse events. Similarly, McLellan et al. reported saving 266 hospital bed days for FLS over usual care in a hypothetical cohort of 1000 patients [15].

Regarding mortality, this study adds to the literature by showing that FLS may improve survival up to 5 years from hip fracture surgery, with the highest protective effect during the first year. In our study, survival curves divide in the early phase and remain almost parallel up to 36 months from baseline, with a favorable trend up to 5 years of follow-up. In a pooled data from 15 studies, Wu et al. found that the FLS significantly reduced the risk of mortality by 3% [12], and then González-Quevedo et al. found a reduction of mortality rate by 5.6% using pre- and post-intervention design [37]. In our study, average mortality rates in the FLS group are similar to those found in the previous research. In contrast, a higher mortality risk was found in the control arm and persisted over the years. Focusing on hip fracture survivors, one out of two functionally independent patients before fracture remains dependent on daily living activities up to 1 year from surgery and never regain their pre-fracture functional level. Compared with matched controls, hip fracture subjects already showed lower independence after 1–2 years in previous studies [44, 45].

This study has several limitations, firstly due to the observational nature of the study design. However, a randomized trial may be considered inappropriate given the poor evidence of equipoise about effectiveness from policy, clinical, and patient perspectives [15]. In the absence of strong evidence about the superiority of FLS-CP, all participants were offered to access the multidisciplinary service; however, they voluntarily chose the more suitable pathway of care according to their preferences and goals. We consider it ethically acceptable based on the principles of CGA and taking into account service’s sustainability issues in a real-world healthcare setting.

Third, we acknowledge the limitations of missing data concerning comorbidities and polypharmacy in U-CP. This reflects the usual orthopedic management mainly focused on surgical outcomes after hip fracture, leaving other care aspects to patients’ GPs. Then, we may have selected patients with different characteristics beyond those measured. Patients able or willing to enter the FLS-CP might be healthier and better educated than those who attend U-CP service, then affecting main outcomes through an healthy user effect bias or unmeasured confoundings, including social, economic, or cultural aspects. However, on one side, the slightly higher rates of delayed surgery (88%) and delayed weight-bearing (23.3%) in the U-CP versus the FLS-CP (78.6% and 8.3%, respectively) could suggest a higher burden of comorbidity. Still, the higher IADL independence in the U-CP compared to FLS-CP before index fracture may substantially attenuate the unhealthy user effect bias hypothesis. These aspects may open the discussion about technical options for hip fracture repair, the severity of osteoporosis, and reasons for delayed weight-bearing, suggesting that people who receive delayed weight-bearing should be considered a different clinical subgroup and deserving a specific evaluation. Fourth, the healthcare facility use may depend on the local service availability and organization to fulfill health-related needs. Therefore, our findings cannot be easily compared with other countries. However, it is conceivable that an orthogeriatric FLS may solve patients’ needs associated with frailty, otherwise undetected in the usual care pathway. Fifth, information bias may be present in the U-CP group about BADL and IADL measures over the follow-up telephone calls. The FLS group was already interviewed about those, while the U-CP group only receives the questionnaire during the telephone follow-up. However, two trained geriatricians performed patients’ and the caregivers’ interviews, reducing potentially over- and underestimating or identifying fallacious memory problems. Fifth, transversal statistical analyses do not allow exploring the causal relationship among variables and outcomes and the 1-year follow-up prevents conclusions about falls and fracture incidence. Although we originally planned to continue the telephone follow-up, after 15 months, we suspended given the decline in patients’ and caregivers’ cooperation. However, the survival analysis was not affected because of the length of follow-up time was assured by accessing the regional administrative registry. The 5-year follow-up time for survival may be a strength of our study. We also recognize that information from CGA was not available in the U-CP group, and functional status was not available in deceased patients over the first-year of follow-up. Finally, the low sample size prevents us from making inferences other than adjustments for age and sex.

In conclusion, patients who enter an interdisciplinary FLS care pathway experience better outcomes regarding adherence to anti-osteoporosis treatments timely initiated after hip fracture repair and healthcare facility admissions owing to adverse events. Older subjects who entered the FLS care pathway tended to be less likely multiple fallers and experienced lower mortality rates starting at the early phase of follow-up. We support the favorable impact of an interdisciplinary FLS programs to bridge the gap in secondary prevention of fragility fracture in high-risk patients.

## Supplementary Information

Below is the link to the electronic supplementary material.

Supplementary file1 (DOCX 19 KB)

Supplementary file2 (DOCX 19 KB)

## Data Availability

On request.
