# Supplementary material for: The interdisciplinary fracture liaison service improves health-related outcomes and survival of older adults after hip fracture surgical repair
Source: Arch Osteoporos. 2022 Oct 17;17(1):135. doi: 10.1007/s11657-022-01171-0 (PMC9576663; doi:10.1007/s11657-022-01171-0)
Supplement: Supplementary file 1 — Supplementary file1 (DOCX 19 KB) [file 11657_2022_1171_MOESM1_ESM.docx]

**Online Resource 1**. Comparison between baseline characteristics of the included patients and those lost to follow-up, according to the Fracture Liaison Service Care Pathway (FLS-CP) or Usual Care Pathway (U-CP)

|  | FLS-CP | | | U-CP | | |
| --- | --- | --- | --- | --- | --- | --- |
|  | Included (*n* = 212) | Lost to FU (n=56) | p-value | Included (*n* = 248) | Lost to FU (n=24) | p-value |
| Women, n (%) | 170 (80.2%) | 40 (75%) | 0.4597 | 183 (73.8%) | 16 (66.7%) | 0.4727 |
| Age (mean ± SD) | 83.8 ± 7.4 | 82.0 ± 8.2 | 0.1671 | 83.7 ± 7.6 | 87.6 ± 8.0 | 0.0072 |
| Pre-fracture BADL, median score (IQR) | 5 (3; 6) | 5 (3; 6) | 0.7237 | 5 (3; 6) | NA |  |
| Pre-fracture BADL independence, n (%) | 129 (60.8%) | 29 (51.8%) | 0.2261 | 121 (59.3%) | NA |  |
| Pre-fracture IADL, median score (IQR) | 3 (0; 7) | 4 (0;7) | 0.9882 | 4 (2; 7) | NA |  |
| - Women | 3 (0; 7) | 4 (0; 7) | 0.628 | 5 (2; 7) | NA |  |
| - Men | 1.5 (0; 4) | 3.5 (1; 5.75) | 0.2897 | 4 (2; 8) | NA |  |
| Pre-fracture IADL independence, n (%) | 82 (38.7%) | 21 (37.5%) | >0.999 | 95 (46.8%) | NA |  |
| Hip fracture type, n (%) |  |  | >0.999 |  |  | 0.8276 |
| - Lateral | 109 (56.8%) | 29 (55.8%) |  | 125 (52.3%) | 11 (47.8%) |  |
| - Medial | 83 (43.2%) | 23 (44.2%) |  | 114 (47.7%) | 12 (52.2%) |  |
| Surgery, n (%) |  |  | 0.7527 |  |  | 0.5718 |
| - Prosthesis | 82 (42.7%) | 24 (46.2%) |  | 93 (38.4%) | 10 (43.5%) |  |
| - Osteosynthesis | 110 (57.3%) | 28 (53.8%) |  | 142 (58.7%) | 12 (52.2%) |  |
| Time to surgery, day (mean ± SD) | 4.1 ± 2.2 | 3.9 ± 2.2 | 0.6808 | 3.9 ± 1.9 | 4.0 ± 1.7 | 0.4597 |
| Surgery within 48 hours, n (%) | 44 (21.4%) | 15 (27.8%) | 0.3616 | 28 (12.0%) | 2 (9.5%) | >0.9999 |
| Weigth-bearing, n (%) |  |  | >0.999 |  |  | 0.6101 |
| - Early | 176 (91.7%) | 48 (92.3%) |  | 152 (76.8%) | 19 (82.6%) |  |
| - Delayed | 16 (8.3%) | 4 (7.7%) |  | 46 (23.2%) | 4 (17.4%) |  |

SD standard deviation, BADL basic activities of daily living, IADL instrumental activities of daily living, IQR interquartile range
