# Supplementary material for: The interdisciplinary fracture liaison service improves health-related outcomes and survival of older adults after hip fracture surgical repair
Source: Arch Osteoporos. 2022 Oct 17;17(1):135. doi: 10.1007/s11657-022-01171-0 (PMC9576663; doi:10.1007/s11657-022-01171-0)
Supplement: Supplementary file 2 — Supplementary file2 (DOCX 19 KB) [file 11657_2022_1171_MOESM2_ESM.docx]

**Online Resource 2**. Baseline characteristics of the FLS-CP participants who underwent CGA.

| Anthropometric parameters |  |
| --- | --- |
| Sex (male) | 42 (19.8%) |
| Age (years) | 85 (79; 89) |
| Weight (kg) | 60 (52; 70) |
| Height (cm) | 160 (155; 165) |
| BMI (kg/m^2^) | 23 (21; 26) |
| Fracture characteristics |  |
| Fracture at hospitalization | 189 (89.2%) first fracture  20 (9.4%) second fracture  2 (0.9%) periprosthetic fracture  1 (0.5%) second femur fracture on a pre-existing fracture |
| Fracture type* | 59 (30.7%) 31.B1  15 (7.8%) 31.B2  10 (5.2%) 31.B3  52 (27.1%) 31.A1  52 (27.1%) 31.A2  4 (2.1%) 31.A3 |
| Surgery | 82 (42.7%) endoprosthesis  110 (57.3%) osteosynthesis |
| Post-fracture independence in ≥5 BADL n (%) | 35 (13.1%) |
| Post-fracture independence in gender-specific IADL n (%) | 24 (9.0%) |
| Load-bearing capacity | 6 (3.1%) grazing  167 (87%) partial  15 (7.8%) out of load  2 (1%) as tolerated  2 (1%) bed/armchair |
| Psycho-physical state |  |
| MMSE test (range 0-30) | 24 (20; 27) |
| NPI test (range 0-20) | 0 (0; 4) |
| Barthel test (range 0-100) | 35 (15; 50) |
| GDS5 test (range 0-5) | 0 (0; 1) |
| IQCODE test | 40 (20.7%) no improving  54 (28%) mild decline  26 (13.5%) moderate decline  73 (37.8%) severe decline |
| CDR test | 137 (64.6%) absent or very mild  23 (10.8%) mild  20 (9.4%) moderate  32 (15.1%) severe, highly severe |
| Parkinson disease | 11 (5.2%) |
| MNA test | 46 (24.7%) malnourished  112 (60.2%) at risk of malnutrition  28 (15.1%) normal nutritional status |
| Comorbidities and Risk factors |  |
| CIRS | 7 (5; 9) |
| Low vision | 88 (41.5%) |
| Hypoacusia | 74 (34.9%) |
| Alcohol consumption (>1 unit/die) | 24 (11.3%) |
| Smoking status | 162 (76.4) non-smoker  39 (18.4%) former smoker  11 (5.2%) smoker |
| Steroid therapy | 17 (8%) |
| Arthrosis | 74 (34.9%) |
| COPD | 30 (14.2%) |
| Rheumatoid/psoriatic arthritis | 13 (6.1%) |
| Hypertension | 130 (61.3%) |
| Atrial fibrillation | 37 (17.5%) |
| Anticoagulant therapy | 185 (87.3%) no therapy  11 (5.2%) warfarin  16 (7.5%) NOAC |
| Depression | 25 (11.8%) |
| Restless legs syndrome | 1 (0.5%) |
| Hypothyroidism | 22 (10.4%) |
| Hyperthyroidism | 3 (1.4%) |
| Diabetes | 32 (15.1%) |
| Stroke | 16 (7.5%) |
| Cardiopathy | 56 (26.4%) |
| Tumor | 22 (10.4%) |
| Previous gastric ulcer | 14 (6.6%) |
| Peripheral vasculopathy | 5 (2.4%) |
| Epilepsy | 5 (2.4%) |
| Medullary canal stenosis/ischemia | 7 (3.3%) |
| Miastenia gravis | 1 (0.5%) |
| AKI | 22 (10.4%) |
| Liver disease/HCV/HBV | 3 (1.4%) |
| Myelodysplasia | 3 (1.4%) |
| Kidney stone | 2 (0.9%) |
| Inflammatory Bowel Disease | 1 (0.5%) |
| Hiatal hernias | 17 (8%) |
| Arterial hypotension | 7 (4.2%) |
| Benign prostatic hyperplasia | 18 (8.5%) |
| Fracture history |  |
| Previous vertebral fracture | 12 (5.7%) one  8 (3.8%) two  4 (1.9%) more than two |
| Previous wrist fracture | 16 (7.5%) one wrist  5 (2.4%) both wrists |
| Previous rib fracture | 6 (2.8%) one  9 (4.2%) two  7 (3.3%) more than two |
| Other previous fracture | 6 (2.8%) pelvis  2 (0.9%) shin  13 (6.1%) humerus  8 (3.8%) malleolus  2 (0.9%) kneecap  4 (1.9%) other |
| Falls during the last year | 22 (10.4%) one  24 (11.3%) two  14 (6.6%) more than two |
| Mineral Bone Density exam | 60 (29%) |
| Initiation antifracture treatment |  |
| Vitamin D and calcium | 207 (97.6%) |
| Specific antifracture treatment | 135 (63.7%) |

*classified according to the Orthopaedic Trauma Association and the AO Foundation by two senior orthopedic surgeons [21]

Dichotomous and categorical data are presented as absolute number and percentage, continuous data are presented as the median and interquartile range

*MMSE* Mini Mental State Evaluation, *NPI* Neuropsychiatric Inventory, *GDS5* Geriatric Depression Scale 5 Items, *IQCODE* Informant Questionnaire on Cognitive Decline in the Elderly, *CDR* Clinical Demential Rating, *MNA* Mini Nutritional Assessment, *CIRS* Cumulative Illness Rating Scale, *NOAC* non-vitamin K antagonist oral anticoagulants, *AKI* acute kidney injury
